# Pharmacologic Approaches for the Management of Apathy in Neurodegenerative Disorders

**DOI:** 10.3389/fphar.2019.01581

**Published:** 2020-01-23

**Authors:** Anamaria Bogdan, Valeria Manera, Alexandra Koenig, Renaud David

**Affiliations:** ^1^Centre Hospitalier Universitaire de Nice, Centre Mémoire de Ressources et de Recherche, Nice, France; ^2^CoBTeK Lab “Cognition Behaviour Technology”, University of Nice Sophia Antipolis, Nice, France

**Keywords:** apathy, amotivation, pharmacology, treatment, Alzheimer’s disease, neurodegenerative disorders

## Abstract

Apathy is one of the most frequent behavioral disturbances in many neurodegenerative disorders and is known to have a negative impact on the disease progression, particularly in Alzheimer’s disease. Therapeutic options are currently limited and non-pharmacological approaches should constitute first line treatments. Pharmacological agents likely to reduce apathy levels are lacking. The objective of the present article is to review recent pharmacological treatments for apathy in neurodegenerative disorders. The Pubmed database was searched with a particular focus on articles published as of January 2017. Current main levels of evidence have been reported so far with cholinergic, glutamatergic and dopaminergic agents to reduce levels of apathy, despite several conflicting results. Treatment duration and samples sizes may have however decreased the validity of previous results. Ongoing studies involving more participants/treatment duration or distinct neural pathways may provide new insights in the treatment of apathy in neurodegenerative disorders.

## Introduction

Apathy is the most frequent behaviorial disturbance in Alzheimer’s disease (AD) and is prevalent in many other neurodegenerative disorders (Husain and Roiser, 2018). Despite apathy is considered a negative symptom in dementia, i.e. a non-demonstrative symptom, it does have negative consequences on the disease progression (Zhu et al., 2019) leading to increased risk of functional disability and institutionalization. The neural correlates of apathy are currently better understood. Brain imaging have shown the involvement of several areas such as anterior cingular and dorsolateral cortex, inferior frontal gyrus (Benoit and Robert, 2011; Kim et al., 2011), and of dopaminergic transmission (David et al., 2008), as well as the involvement of fronto-subcortical circuits (Riveros et al., 2018). Additionally, severity of apathy has been associated with lower CSF Aβ_42_ concentrations in Alzheimer’s disease (Vergallo et al., 2019). In this line, depression, that is often misdiagnosed with apathy, likely involves different structures (prefrontal orbitofrontal cortex (Lavretsky et al., 2007), cingulate, thalamus) (Starkstein et al., 2009; Zahodne et al., 2013), and neurotransmission pathways (5-HT transmission reduction in posterior cingulated and amygdala-hippocampus complex) (Benoit and Robert, 2011).

Considering the important negative impact of apathy in the evolution of AD, therapeutic options are needed. Current therapeutic treatments mainly rely on non-pharmacological approaches (Mueller et al., 2018). Moreover, conventional psychotropic drugs often overprescribed in AD, such as antipsychotics and Selective-Serotonin Reuptake Inhibitors (SSRI) antidepressants may increase levels of apathy in neurodegenerative disorders and may have overall insufficient effect to alleviate levels of BPSD (Anand et al., 2018). Weighing risks and benefits, it is recognized that psychotropic agents should be prescribed with caution in dementia (Azuar and Levy, 2018; Phan et al., 2019).

Previous articles have been published, including several randomized controlled trials (RCT), but pharmacological therapeutic options for the management of apathy are currently limited.

The present article makes a review of current pharmacological approaches available for the management of apathy in AD and related disorders.

## Method

Previous reviews investigating management options for apathy have been recently published. Therefore, we focused our research on published articles as of 2017.

We searched the Pubmed online database between January 1^st^, 2017 and May 1^st^, 2019, for articles published in English, using the following method and keywords:

(((((apathy[Title/Abstract]) OR amotivation[Title/Abstract]) OR abulia[Title/Abstract])) AND ((treatment[Title/Abstract]) OR pharmacological intervention[Title/Abstract])) AND (((((((alzheimer[Title/Abstract]) OR vascular dementia[Title/Abstract]) OR mixed dementia[Title/Abstract]) OR lewy body[Title/Abstract]) OR parkinson[Title/Abstract]) OR dementia[Title/Abstract]) AND (“2017/01/01”[PDat]: “2019/12/31”[PDat])).

All abstracts were screened by two reviewers in order to assess their relevance to the topic.

**Figure 1:**
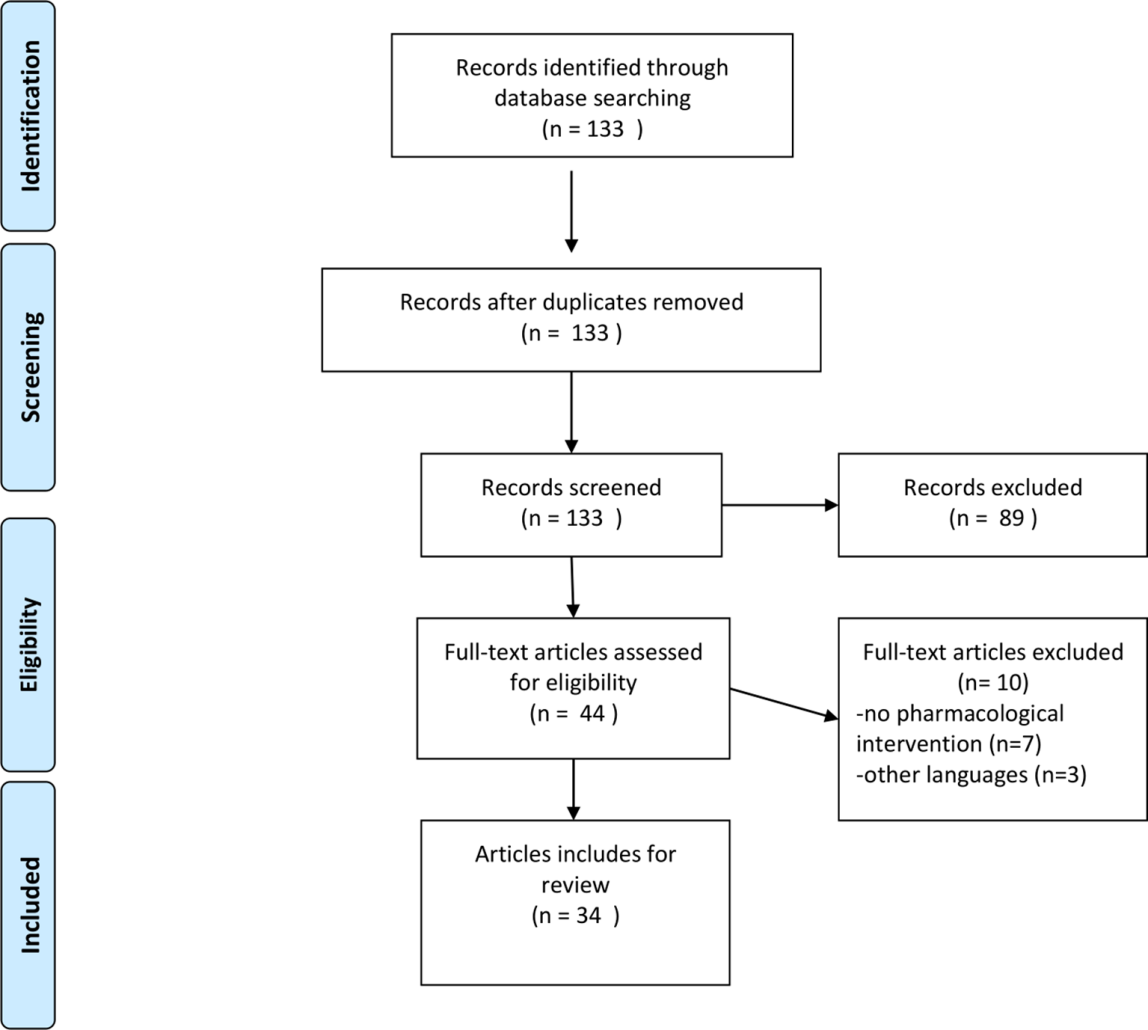
Study flow diagram.

See **Figure 1** for the study flow diagram

## Results

Results are presented according to their level of evidence, respectively in Alzheimer’s disease and in other neurological and/or neurodegenerative disorders. Main results are summarized in **Table 1**.

**Table 1:** Summary of recent drug trials targeting apathy in different neurodegenerative disorders.

Study		Journal	n	disorder	Intervention	Treatment duration	Outcome and results	Comments
Authors	Study design							
Finger et al., 2018	RCT, Phase 1 + phase 2	Ongoing study	Total n = 60	FTD	Intranasal oxytocin different dosage between phase 1 and 2	18 weeks, phase 1, 18 weeks, phase 2 36 weeks	NPI Apathy-domain	Results not yet published
Scherer et al., 2018	RCT Phase 2	Ongoing study	200	AD	Methylphenidate 20mg/day	6 months	mADS-CGIC NPI DAIR	Results not yet published
Zhou et al., 2019	RCT	Experimental and Therapeutic Medicine	80 40/40	AD	Memantine 20 mg + Citalopram 30 mg/day vs Memantine 20 mg/day + placebo	12 weeks	NPI Apathy-domain: Reduction of apathy, p = 0,002	Memantine + citalopram superior to memantine + placebo
Gelderblom et al., 2017	RCT	PLOS One	40 20/20	Huntington	Bupropion 300 mg/day	10 weeks	Apathy primary outcome AES-I not efficient p < 0.05	
Lhommee et al., 2018	Open label randomized trial	The Lancet Neurology	251 127/124	PD	L-Dopa + dopa agonist/L-Dopa + dopa agonist + DBS The L-dopa equivalent daily dose increased by 21% (mean change 245·8 mg/day in patients allocated medical therapy alone) and decreased by 39% (–363·3 mg/day in those assigned DBS + medical therapy	2 years	NPI apathy-domain And Starkstein Apathy Scale	Apathy scores worsened in both groups, higher with DBS
Auffret et al., 2017	Observational study	Journal of the Neurological Sciences	12	PD	Apomorphine pump mean daily dose 57.7 ± 27.0 mg/day	6 months	Apathy: improvement on LARS- I P < 0.05	Patients advanced Parkinson’s disease and contraindication to DBS
Oshawa et al., 2017	Observational	Journal of Alzheimer’s Disease Reports	20	AD	ninjin’yoeito	12 weeks	NPI Significant improvement in Apathy after 4weeks P < 0.001	multicomponent drug, several effects including dopamine modulation
Nagayama et al., 2019	Open label	Journal of the Neurological Sciences	30	PD	Istradefylline, 40 mg	12 weeks	Apathy scale: significantly reduced at the 2^nd^, 4^th^, 8^th^, 12^th^ w p = 0.02, 0.05, 0.01 and 0.005	Istradefylline = adenosine A2 receptor antagonist
Moretti et al., 2017	Open label, observational	Parkinson’s Disease	48	Parkinson’s dementia	Rivastigmine 9.5 mg/24h transdermal patch Add-on therapy	12 months	Apathy primary outcome, AES-S, AES-C, sub-item of NPI No efficacy	

### Alzheimer’s Disease

#### Reviews and Meta-Analyses

One recent article from January 2017 reviewed recent pharmacological and non-pharmacological approaches for the management of apathy in AD (Theleritis et al., 2017) and reported the potential interest of cholinesterase inhibitors (ChEIs), methylphenidate and gingko biloba in reducing levels of apathy, whereas Sepehry et al’s review did not reveal any significant treatment effect likely to reduce apathy in AD (Sepehry et al., 2017). In Theleritis, among 6 studies using galantamine (4 RCT and 2 open-label studies), five studies did show an improvement of apathy levels after treatment. Rivastigmine (8 open-label studies) showed improvements in apathy levels in all studies. Memantine (3 RCT, 1 open-label study, and 1 post marketing surveillance study) improved apathy in 4 studies. In the meta-analysis of Kishi (Kishi et al., 2017), memantine was not superior to controls for the management of negative symptoms, including apathy, in AD.

In Ruthirakukan’s meta-analysis (Ruthirakuhan et al., 2018), methylphenidate (3 studies), compared to placebo, was likely to reduce apathy in AD, depending, however, on the assessment interview used (AES versus NPI-apathy domain).

In a review published in 2017, Lanctot et al. concluded that progress has been made in the phenomenology, the neurobiology and the treatment of apathy. Regarding the pharmacological treatment, they found evidence that ChEIs and memantine improve apathy, whereas ChEI withdrawal can worsen apathy. Modafinil improved apathy in 2 case reports, but a small RCT reported no significant improvement in apathy over 8-week treatment in AD. Methylphenidate was effective for apathy in mild to moderate AD as reported in 3 placebo-controlled trials (Lanctot et al., 2017).

#### Randomized Controlled Trials (RCT)

In the ongoing ADMET2 (Apathy in Dementia Methylphenidate Trial) phase III RCT study, authors reported the interest of using methylphenidate (Scherer et al., 2018) and planned to include 200 AD individuals (20 mg/day methylphenidate for 6 months and apathy assessed using the NPI apathy-domain). Results are expected as of 2020. The previous ADMET study showed a benefit of using methylphenidate after a 6-week treatment period among 60 participants (Rosenberg et al., 2013).

A memantine (20 mg/day)-citalopram (10 to 30 mg/day) combination (vs memantine + placebo) was significantly associated with a reduction of apathy in a group of AD individuals over a 12-week treatment period, using the NPI apathy-domain as outcome measure (Zhou et al., 2019).

In the ASCOMALVA study, comparing a subgroup of AD individuals (n = 56) receiving a combination of donepezil and choline alphoscerate (a cholinergic precursor) versus a subgroup (n = 57) with donepezil alone (24-month treatment period), the combined treatment was more effective on apathy levels than donepezil alone (Rea et al., 2015; Carotenuto et al., 2017).

#### Observational Studies

One observational study with AD individuals using ninjin’yoeito, a multicomponent drug with several effects including dopamine modulation, apathy (using the NPI apathy-domain) was significantly improved as of 4 weeks after treatment initiation (Ohsawa et al., 2017).

#### Animal Research

In a mice model of AD, the use of melatonin for 6 months (10 mg/kg) was efficient in reducing levels of apathy and anxiety as well amyloid and tau burden in transgenic AD mice, and could thus constitute a promising treatment opportunity in human research (Corpas et al., 2018).

### Other Disorders

#### Parkinson’s Disease

In several other neurodegenerative diseases, such as Parkinson’s disease (PD) and Dementia with Lewy Body (DLB), dopaminergic agents, and cholinesterase inhibitors (ChEIs) have demonstrated an interest in reducing levels of apathy (Liu et al., 2019), but therapeutic options remain limited for the general management of nonmotor symptoms in PD, including apathy (Seppi et al., 2019). In an open-label observational study, targeting apathy as primary outcome (measured with NPI and AES), patients received transdermal rivastigmine (9,5 mg/day), one of the current available ChEIs. Rivastigmine did not improve apathy over a 12-month period (Moretti et al., 2017)

In Liu’s meta-analysis, authors reviewed 19 articles regarding the pharmacological treatment of apathy in PD and 4 articles in DLB. In the selected studies, apathy was either the 1st or a 2^nd^ outcome. In the PD population, 13 articles were RCT, 4 open-label studies, 1 case series and 1 case report. The Apathy was measured using different scales: apathy scale, the Non-Motor Symptoms Scale (NMSS), the Neuropsychiatric inventory (NPI), Lille Apathy Rating Scale (LARS) and Unified Parkinson’s Disease Rating Scale (UDPRS) for PD, and the NPI for DLB.

The investigated drugs were: rotigotine, piribedil, methylphenidate, rivastigmine in double-blind placebo-controlled studies. Rotigotine, a dopamine agonist, improved apathy in all 4 RCTs. Piribedil, a D2 and D3 receptor agonist with alpha2- adrenergic antagonist properties, reduced apathy scores in PD patients who developed apathy after deep brain stimulation. Methylphenidate, a dopamine norepinephrine reuptake inhibitor, reduced apathy by 7 points on LARS compared to only 1-point reduction with placebo among 12 patients.

Rivastimine (ChEIs) reduced apathy on the LARS (8,5 points) compared to placebo (0,2-point reduction) in a 30-patient study. Five other RCT that assessed the pharmacological treatments of apathy in PD were analyzed. Rasagiline, an MAOI-B(monoamine oxidase B inhibitor) and atomoxetine, a SSNRI (selective serotonin-norepinephrine reuptake inhibitor), did not improve apathy levels. Decosahexaeonic acid (DHA), an omega-3 fatty acid, was not superior to placebo in the treatment of apathy. Amantadine, an agonist at many different receptors, decreased apathy levels by 0,9 compared with a 0,7 increase in the placebo group. For open-label studies, out of which only one included a control group, authors showed an improvement of apathy with galantamine on the NPI while rivastigmine did not decrease NPI-assessed apathy symptoms. A traditional Japanese medicine, yokukansan, significantly improved NPI apathy scores in 23 patients, but the exact scores were not reported.

Pump-based Parkinson therapies using apomorphine (non-selective dopamine agonist) infusion and levodopa-carbidopa intestinal gel allowing a more continuous dopaminergic stimulation tend to reduce several nonmotor symptoms in PD such as sleep, mood disorders, and apathy (Mundt-Petersen and Odin, 2017). Effects of add-on apomorphine in advanced PD were significant on apathy (assessed using the LARS-i) after 6 months of therapy (Auffret et al., 2017).

In late stages of PD, L-dopa showed significant improvements in levels of mood/apathy (Rosqvist et al., 2018). The use of Levodopa in individuals diagnosed with Parkinson’s disease did not show significant reduction of apathy in the EARLYSTIM trial (Lhommee et al., 2018).

In an open-label trial including 30 PD individuals, the use of istradefylline (adenosine A2A receptor antagonist), an anti-parkinsonian agent, significantly reduced levels of apathy (assessed using the Apathy scale) after a 12-week treatment period (Nagayama et al., 2019).

In the meta-analysis, including recent RCT, from Wang et al. (2018), transdermal rotigotine significantly improved apathy in PD individuals.

In another recent review, dopamine agonists (piribedil, rotigotine and pramipexole) have been reported to improve levels of apathy in PD (Rektorova, 2019).

#### Fronto-Temporal Dementia (FTD)

Intranasal oxytocin for the management of neuropsychiatric symptoms in FTD is currently under trial (Finger et al., 2018). In this ongoing study, 60 individuals diagnosed with FTD will be included for a 6-week treatment period with oxytocin. Oxytocin is a neuropeptide, synthetized by magnocellular neurons in the hypothalamus (paraventricular and supraoptic nuclei), with behavioral effects in animals and humans. It has only one known receptor, OXYR, widely distributed throughout the brain (Lee et al., 2009). The use of psychostimulants may help to decrease levels of apathy in FTD (Young et al., 2018).

#### Huntington Disease

In Huntington disease, the use of bupropion (a norepinephrine dopamine reuptake inhibitor) did not show a significant effect in reducing levels of apathy, globally or by domain (cognitive, behavioral, and emotional) (Gelderblom et al., 2017). This RCT targeting apathy as primary outcome (using the Apathy Evaluation Scale AES) over a 10-week period of treatment with bupropion (150 or 300 mg/day vs placebo).

#### Other Disorders

One RCT investigating nefiracetam (a nicotinic, cholinergic, and NMDA receptor activity enhancer) was found relevant in reducing apathy in a post stroke population. Results were however not statistically significant at week 12 and only a limited number of individuals were included (n = 13) (Starkstein et al., 2016).

Aragona et al, however, reported an improvement in apathy levels (AES) with bupropion in a case report of post-stroke induced-apathy (left thalamus hemorrhagic stroke) (Aragona et al., 2018).

## Discussion

**Figure 2:**
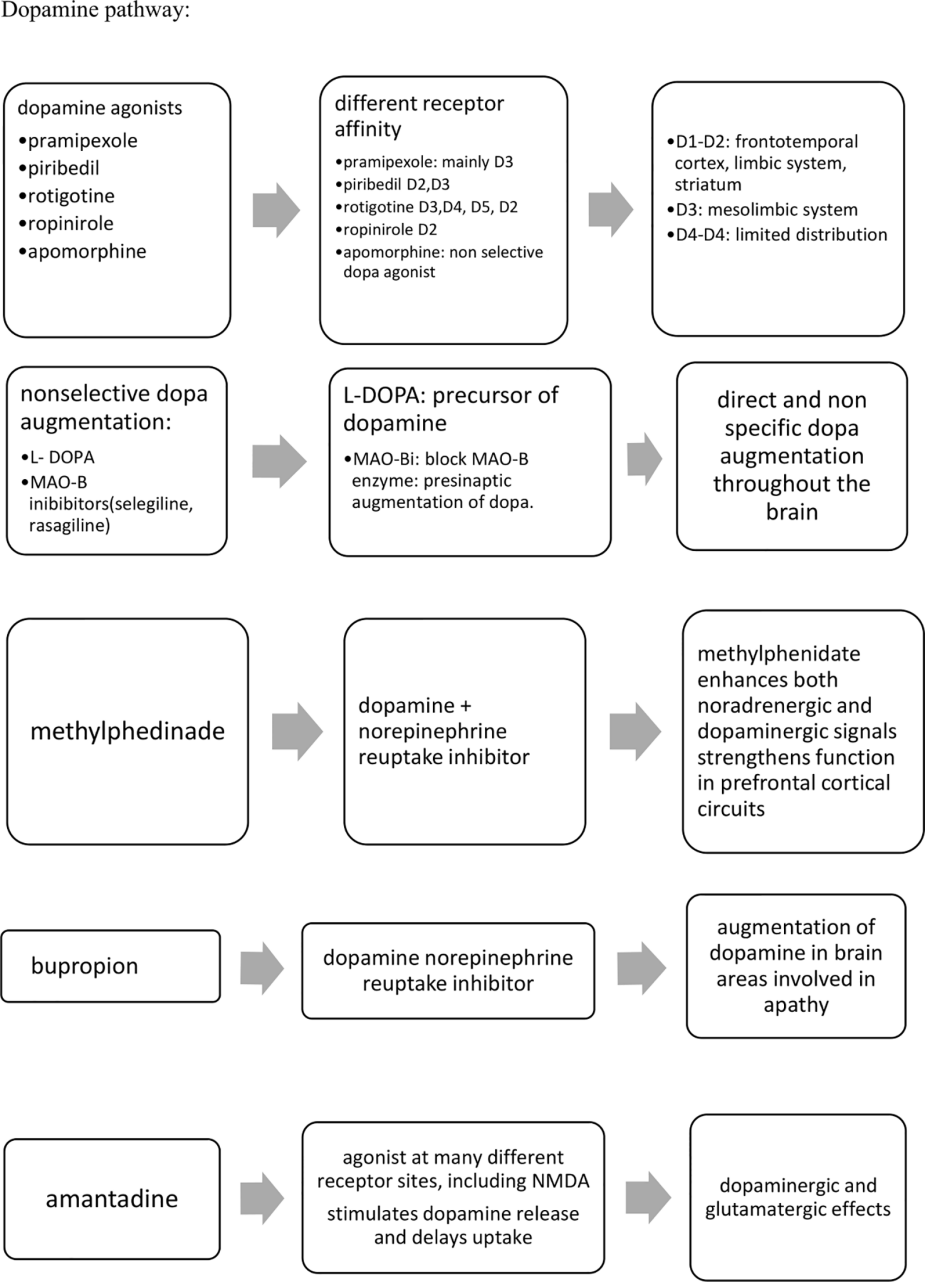
Summary of pharmacological agents with higher levels of evidence for the management of apathy.

Despite the early and important prevalence of apathy in many neurodegenerative disorders, available pharmacological treatments remain currently limited. Non-pharmacological approaches have to be considered first line treatments for apathy as for all behavioral and psychological symptoms in dementia, eventually in association with pharmacological agents if required. To our knowledge and irrespective to the putative neural pathway, no pharmacological agent is currently recommended for the specific management of apathy in neurodegenerative. Investigated agents still remain so far off-label prescribing. Drug trials on apathy have mainly focused individuals with AD and PD. Despite conflicting results, agents involved in the cholinergic neural pathway (ChEIs), alone or in association with another pharmacological agent (cholinergic precursor) or glutamatergic agent (memantine) (alone or in association with SSRI) seems to be efficient in reducing levels of apathy, even for an extended treatment period. Agents having effects on the dopaminergic neural pathway (methylphenidate and bupropion, that inhibit the recapture of dopamine, dopamine agonists, L-Dopa) have also shown benefits in reducing apathy, but most of published results often included a limited number of participants over a limited treatment period. Results from the ADMET2 ongoing study (200 participants over a 6-month treatment duration) will likely provide insights on the interest of using dopaminergic agents for the treatment of apathy. The **Figure 2** summarizes the main neural pathways targeted so far for the treatment of apathy. Results from drug trials investigating distinct hypotheses such as oxytocin are also expected.

All aforementioned pharmacologic options for apathy are however likely to be associated with possible side effects that have to be taken into account when prescribed. Considering the cholinergic pathways, main side effects using CHEIs are digestive (nausea, diarrhea, vomiting), cardiogenic (mild decrease in the number of heart beats, rhythm disorders), and neuropsychologic (hallucinations, agitation, aggressiveness, seizure, fatigue, cephalalgia). With glutamatergic agents (memantine), the following side effects have been reported: fatigue, cephalalgia, digestive symptoms (nausea, constipation, vomiting), neuropsychologic symptoms (anxiety, hallucinations, sleep disorders, excessive sleepiness, confusion). Regarding dopamine-targeting agents, reported side effects are as follows, respectively with methylphenidate (addictive behaviors; amphetamine-like intoxication including hypertension, tachycardia, agitation, delusion, seizure,…; cardiogenic symptoms such as arrythmia and hypertension), with L-Dopa and dopa agonists (nausea and vomiting, anorexia, hypotension, excessive sleepiness or nightmares, anxiety, agitation, delusion, compulsive behaviors, and hypersexuality, dyskinesia).

Additionally, alternative therapeutic approaches such as transcranial direct current stimulation (tDCS) may constitute new options for the treatment of apathy, considering the fact that such techniques enable the possibility to directly stimulate deep cerebral structures (anterior cingular cortex for apathy). One study investigated this hypothesis with negative results on apathy (40 AD individuals with 6 tDCS sessions over 2 weeks) (Suemoto et al., 2014). An optimized design with an increased and repeated number of sessions would probably be of interest.

On a more clinical point of view, apathy is sharing several overlapping symptoms with depression that could lead to inappropriate diagnoses, and thus consequently to an inappropriate treatment prescribing. Despite distinct neural pathways (Murakami et al., 2013; Eyre et al., 2017; Prange et al., 2019), apathy and depression are sharing similar symptoms such as diminished interests, psychomotor retardation, diminished decision making and initiatives. Apathy is frequently misdiagnosed with depression leading to antidepressant prescribing. In this line SSRIs (as well as SSNRIs selective serotonin and norepinephrine reuptake inhibitors such as duloxetine), usually prescribed as first line therapy in depression, might increase the severity of apathy, when inappropriately prescribed for apathy symptoms, whereas they are effective in reducing depressive symptoms (in a Parkinson’s disease population) (Takahashi et al., 2019). Several authors suggest that the use of monoamine oxidase inhibitors (instead of other antidepressant drugs) should be initiated first in chronic neurodegeneration (Riederer and Muller, 2017).

Considering evidences from non-neurodegenerative diseases, others pharmacologic approaches showed benefits in reducing apathy-like behaviors (improvement of negative symptoms with aripiprazole, antidepressants, or topiramate in schizophrenia (Veerman et al., 2017).

Additionally, recent animal studies showed interests in using different pharmacologic targets such as antagonist of muscarinic acetylcholine receptors (Hailwood et al., 2019) or selective 5-HT2C receptor ligand (Bailey et al., 2018) to enhance amotivation and goal-oriented behaviors.

However, despite the different proposed approaches, the management of apathy and apathy-like behaviors remains challenging in daily clinical routine.

## Author Contributions

AB and RD participated in the review of the literature and the manuscript’s writing. VM and AK participated in the manuscript’s writing and editing.

## Conflict of Interest

The authors declare that the research was conducted in the absence of any commercial or financial relationships that could be construed as a potential conflict of interest.
